# The role of artificial intelligence in plain chest radiographs interpretation during the Covid-19 pandemic

**DOI:** 10.1259/bjro.20210075

**Published:** 2022-05-26

**Authors:** Dana AlNuaimi, Reem AlKetbi

**Affiliations:** ^1^ Westford University-UCAM, Sharjah, United Arab Emirates; ^2^ Dubai Health Authority, Dubai, United Arab Emirates

## Abstract

Artificial intelligence (AI) plays a crucial role in the future development of all healthcare sectors ranging from clinical assistance of physicians by providing accurate diagnosis, prognosis and treatment to the development of vaccinations and aiding in the combat against the Covid-19 global pandemic. AI has an important role in diagnostic radiology where the algorithms can be trained by large datasets to accurately provide a timely diagnosis of the radiological images given. This has led to the development of several AI algorithms that can be used in regions of scarcity of radiologists during the current pandemic by simply denoting the presence or absence of Covid-19 pneumonia in PCR positive patients on plain chest radiographs as well as in helping to levitate the over-burdened radiology departments by accelerating the time for report delivery. Plain chest radiography is the most common radiological study in the emergency department setting and is readily available, fast and a cheap method that can be used in triaging patients as well as being portable in the medical wards and can be used as the initial radiological examination in Covid-19 positive patients to detect pneumonic changes. Numerous studies have been done comparing several AI algorithms to that of experienced thoracic radiologists in plain chest radiograph reports measuring accuracy of each in Covid-19 patients. The majority of studies have reported performance equal or higher to that of the well-experienced thoracic radiologist in predicting the presence or absence of Covid-19 pneumonic changes in the provided chest radiographs.

## Introduction

Coronavirus disease 2019 continues to spread worldwide giving rise to critical challenges for both the public health and the medical communities. The Beta-corona virus also known as severe acute respiratory syndrome coronavirus 2 (SAR-coV-2) was first reported in Wuhan, China in December 2019 and is believed to have a zoonotic origin. It is essentially transmitted by contact with respiratory droplets and has an incubation period of 2–14 days. The majority of patients develop mild symptoms such as fever and dry cough. However, patients can develop fatal complications such as severe bilateral pneumonia and acute respiratory syndrome, multiple organ failure and septic shock. This is seen more in males with a percentage of approximately 54.3% and a median age group of 56 years old. Intensive care unit admissions are usually seen in patients older in age and those with multiple co-morbidities.^1,2^

Urgent efforts for the early detection of patients, isolation, adequate treatment and contact tracing were initiated. Global efforts to contain misinformation and to also reduce the huge economic impact were applied. The World Health Organization’s confirmed cases up to the current date is estimated to be **492,198,439** with the total number of deaths reaching **6,159,474**. This has led the healthcare organisations to face tremendous pressure in coping with the rapidly increasing number of patients.^1,2^

AI is promptly evolving in every aspect of the healthcare systems and especially in the field of diagnostic radiology. During the current pandemic, AI has played a significant role in the early detection and diagnosis of Covid-19 patients, it aided in the monitoring of the given treatment and predicting mortality rates, as well as in the tracing of contacts of Covid-19 patients, the development of new medication and vaccine for the virus and finally the reduction of overall workload on healthcare workers.^3^

The role of AI in diagnostic radiology is expanding as part of the routine workflow in the radiology department especially with the current pandemic thus radiologists need to be familiar to its underlying current concepts and its terminology. It aids in extracting more information from the various diagnostic radiology studies ranging from plain radiography to computed tomography and magnetic resonance imaging, including newer hybrid imaging studies such as positron emission tomography computed tomography (PET-CT). It can also help in the detection of the highest probability of disease from the data available, for example, the detection of a pulmonary nodule or opacities on a chest radiograph or chest CT. Another feature of AI is the automated lesion segmentation which aids in calculating the total tumor volume, for example, in hepatocellular carcinoma by using boundary definition. Another example is the classification of chest CT angiography into positive or negative for having a pulmonary embolism.^4^

AI is defined as the computerized machines imitation of the human intelligent behavior. It is the development of such computerized algorithms which has enabled it to accomplish human tasks giving it the ability to learn and to solve problems. It comprises of the use of machine learning, deep learning, artificial neural networks and radiomics. Machine learning deploys features that necessitates the classification of the given data. The more data that are made available, the higher the performance of the given algorithm. Deep learning has the capability to enable the representation of the given data in multiple layers of its abstraction for example its texture or complex shape. Deep learning, which is subset of machine learning, has a great impact and is currently pioneering in image recognition tasks in which it can recognize the complex patterns in any imaging data giving an automated pattern to a quantitively radiographic value. Neural networks operate to correctly guess and compare between the given data. Imaging data is readily available and with the addition of information from the clinical outcomes the use of radiomics is created which has a great value in medical decision-making and risk stratifications of many different diseases including cancers.^5,6^

In comparison with the radiology-trained physicians who asses the images visually in a more qualitative manner, the recent deep-learning methods has equalled or is even superior to the human ability in these task given applications. In deep learning, a previous definition by humans is unnecessary therefore this lowers the pre-processing steps hence increasing the speed of the automatic identification of the diseased tissues. The introduction of AI in diagnostic radiology aids in elevating the patients quality of care, increasing the patients safety, and shortening time for radiology report generation. Furthermore, AI appears to play a crucial role in thoracic imaging during the Covid-19 pandemic by providing diagnostic reports at a faster rate thereby decreasing the overwhelming stress faced by the radiologists from the heavy workload.^3,5–7^

### Covid-19 pandemic : The role of thoracic imaging

Covid-19 viral pandemic has rendered an unprecedented worldwide challenge to all healthcare systems as well as a devasting impact on societies and on world economies. In March 2020, the World health organization declared Covid-19 as a global pandemic.^8^ A worldwide strain on healthcare systems occurred with massive shortages in crucial protective equipment and in the presence of qualified healthcare providers.^9^ Respiratory failure can occur in severe Covid-19 pneumonia and is rapidly fatal in 2–8% of patients especially in elderly with underlying medical conditions such as cardiovascular diseases, chronic respiratory diseases, diabetes mellitus and hypertension. Non-pulmonary diseases such as hepatic, renal, neurological diseases and patients with coagulopathies also have a higher prevalence of complications.^2,9,10^

The main method of diagnosis which remains the gold standard up to the current date is the reverse-transcription polymerase chain reaction RT-PCR laboratory test taken by nasal swabs. Nonetheless, it has a sensitivity of 60–70% only and is non-readily available due to limitations of resources in some regions.^8,10,11^ Its results are highly dependent on the site where the swab is taken, the expertise of the medical personnel taking the swab and the viral load at the time of the swab. It is also considered time-consuming as the results are available only after 6 to 9 h.^12^

Plain chest radiography is the most common radiological examination in the emergency department setting.^13^ Chest radiography and CT have a secondary role in the diagnosis of Covid-19 patients.^10^ There are several studies currently published where chest CT was found to have a higher sensitivity than the PCR swab and was recommended to be considered as a diagnostic tool for Covid-19 infection detection.^14^ Nonetheless, the American College of Radiology recommends chest CT only for patients who are symptomatic and have specific clinical indications.^15,16^

The major challenge faced by the healthcare systems in the Covid-19 pandemic is the timely establishment of the diagnosis and monitoring of the disease progression.^2,8^ During the pandemic, an approximation of 20% of chest radiographs were reported by general radiologists as normal while they were found to be abnormal after a second peer reading.^11^ Nevertheless, only 65% of positive finding on chest radiographs were picked up by expert radiologists as Covid-19 pneumonic changes usually appear as subtle findings in the majority of patients.^12^ This can be explained by the limited number of experienced thoracic radiologists and the sudden increase in work overload during the pandemic.^11^

A few studies on the role of chest radiography proved it to be non-specific for Covid-19 pneumonia detection as findings can be seen in other viral infections, aspiration pneumonia and drug toxicity reactions thereby it alone has low sensitivity and specificity for the disease.^8,17^ The typical abnormalities seen on the images include peripheral ground glass opacities, bilateral lower lobe consolidation and diffuse air space disease. Rare findings include pneumothorax, pleural effusion and lung cavitation.^14,18,19^ Patients who are PCR positive and are asymptomatic usually present with a normal chest radiograph.^12^

Chest radiographs are insensitive in early and mild Covid-19 infections and are usually used as a first line tool in the triage of patients in the emergency department.^18,20^ Nevertheless, chest radiography has an advantage over computed tomography of being readily available, cheaper, portable with a lower radiation dose. They also aid in the assessment of the disease progression and in detecting other diseases contributing to the patients symptoms.^11,20^

In a study done by Wong at al., it was noted that out of all the Covid-19 positive patients who required hospitalization almost 69% actually had abnormal chest radiograph findings and during the hospital stay almost 80% of the patients developed abnormal chest radiographs and the findings were usually seen most extensively at day 10 of the disease progression.^19^ Nonetheless, the visual evaluation by the radiologist for subtle finding presents as a challenge and is time-consuming.^21^

Chest CT was used at an unprecedented scale during the Covid-19 global pandemic and is superior to chest radiography is detecting early disease even in PCR negative and asymptomatic patients; however, it has a much higher radiation dose, is more costly and takes longer infection control measures.^9,11,12,14^

### Artificial Intelligence in Covid-19 chest imaging

AI was first described in 1956 and is a rapidly evolving field of computer science set to impact every aspect in the healthcare systems. Machine learning uses algorithms to aid in image classification and segmentation relying on object recognition. Segmentation means the separation of the lesion from its adjacent tissue which is crucial for image analysis in covid-19 patients. Deep learning uses deep neural networks that were trained with large data. The image recognition systems train when given sets of examples and later on adjust their parameters to adapt to the new input data this is also called autonomous feature learning.^8,11^

Computed Vision uses deep learning algorithms called deep neural networks to extract hidden and valuable structures from the provided raw data. Convolutional neural networks (CNN) aid in the classification of the images. However, CNN has the drawback of its performance estimation based on an exaggerated data sets thus the greater the provided data to train the algorithm the higher the generalisation power.^12^

Nonetheless, there can be a subject of bias in diagnostic imaging due to the quality and quantity of the provided radiological images for training such as inequality in the number of images with positive and negative findings, the difference in image characteristics such as the kilovolts, milliamperes, pixel and size of the images as well as the presence of labels, medical devices such as tubes, wires and pacemakers as well as other causes of artifacts. These can be prevented by using an external set for the evaluation of the trained models, the segmentation of the lung regions in thoracic imaging and pre-processing of the images to decreases distortion and artefacts.^12^

**Figure 1.**
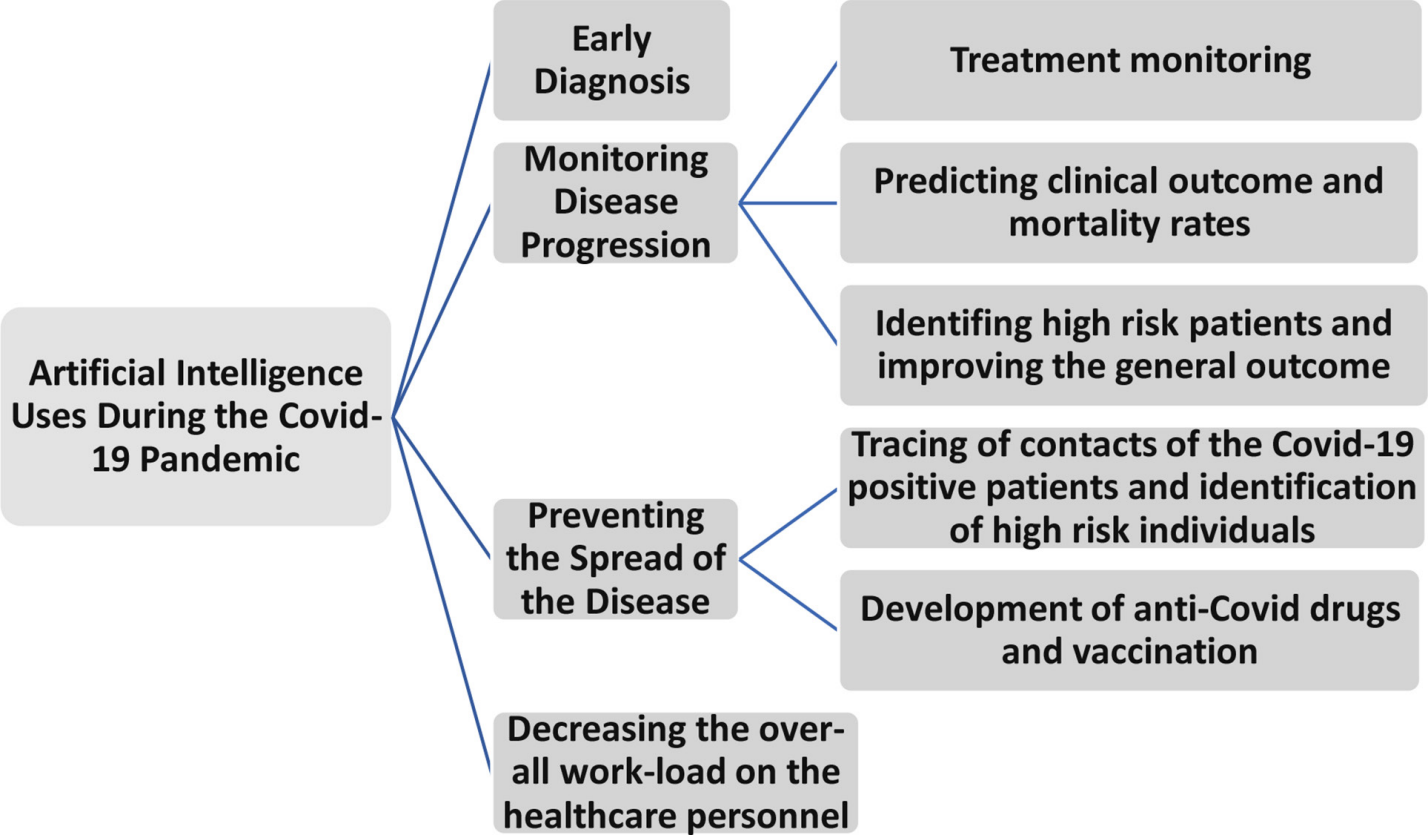
Horizontal chart showing the uses of AI in healthcare during the covid-19 pandemic.

During the Covid-19 pandemic, the use of AI has accelerated especially in healthcare where it aided in analysing clinical data, early detection of the disease, treatment monitoring and prevention of the spread of the disease as demonstrated in Figure 1.^3,22^

It can also be used to predict the need for hospital admission, the length of the hospital stay and the risk of patients developing a critical outcome. Early diagnosis and the initiation of the prompt treatment is crucial in reducing the risk of mortality of Covid-19 patients. Furthermore, the use of AI in chest imaging can identify the high-risk patients and thereby improve the general outcome.^23^

AI aided in the increase in speed of reaching the diagnosis and ruling out other causes of pneumonias with an objective quantification leading to a decrease in subjectivity and variability of the diagnosis. Improving the overall workflow for radiologists by shortening the reading times by 65%.^11,20^ There are three main areas of interest in research on the use of AI in Covid-19 diagnostic imaging which can be explored: First is binary diagnosis predicting whether Covid-19 infection is positive or negative. Second is the segmentation and the quantification of the ground glass lung opacifications seen in the images and finally is the ability to distinguish between Covid-19 pneumonia from other types of pneumonias.^10^

In a study done in 2020 by Andrew Borkowski using Microsoft’s Custom Vision AI algorithms (www.cutomvision.ai) which was trained by Covid-19 datasets, the chest images were uploaded into the website with which the machine learning AI gave the probability of Covid-19 pneumonia in the patient. The study yielded a sensitivity of 92.9% of positive predictive value, 94.8–98% Covid-19 pneumonia, 89–91.8% of other types of pneumonia and 88.8–95% of normal lung parenchyma. Such algorithms can aid in the rapid screening and triage of Covid-19 patients as well as in providing a second opinion to the radiologists report.^8^ A few other software tools as well as Covid-19 chest radiograph datasets are available online for free use in research.^10,24^

An article by Van Ginneken comparing three types of available AI algorithms by using the area under the curve where the equivalence of the chance of a random Covid-19 positive image receiving a higher score than a random negative one was used and all three algorithms showed a promising results. DeepCOVID XR (AUC = 0.88), CV 19-Net (AUC = 0.94) and CAD4COVID X-ray (AUC = 0.81) compared to a senior radiologist in which the AUC was 0.85.^20^

A recent retrospective study by Zhicheng Jiao at al. in which the use of AI in chest radiography in addition to the patient’s clinical data was used for the prediction of the prognosis and the progression of the disease was assessed. The binary outcome of the disease severity was classified into critical or non-critical and the results compared to a severity score used by radiologists. It concluded that the application of AI deep learning in chest radiographs is superior to using clinical data combined with the radiologists scoring. However, there were several limitations to the study such as a lower performance in external testing in comparison with internal testing making generalisation more difficult, the rapid disease progression in several patients giving a short timeframe to study and its determination by critical events such as hospitalisation.^23^

Another retrospective study by Esposito et al in which 301 PCR positive patients were given the qualitative chest radiograph radioscore by two qualified thoracic radiologists into mild, moderate and severe then combined the clinical and laboratory data to predict the clinical outcome of the patients. Radioscore proved a reliable tool by the AI evaluation to be used as a predictor of the clinical outcome.^18^

Other studies have shown a superior automatic classification of images in chest radiography than in chest computed tomography where the average accuracy was noted to be 96% in comparison with that of the chest CT of 90%.^12^

In a study done by Wu et al, an AI algorithm was employed in reporting chest radiography and was compared to five year three radiology residents where the algorithm was trained on 342 images, of which 126 were emergency department chest images and concluded that there is no significant difference in sensitivity and specificity between the two except for the positive predictive value which was found to be higher with the AI algorithm with an overall mean AUC of 0.807. The radiology residents seemed to perform better in diagnosing subtle anomalies such as masses or nodules, misplaced tubes and lines and different forms of consolidation while the AI algorithm was better in the detection on non-anomalies findings such as the presences of tubes and lines, obvious visible anomalies such as pleural effusion, cardiomegaly and pulmonary edema. Therefore, well-trained AI algorithms can be used for preliminary reporting of plain chest radiographs which can be later on corrected or approved by the attending radiologist to avoid missing any potential important findings and is seen to improve overall accuracy of the reports and expedite radiology reads by providing a second reading as well as to address the scarcity of radiologists and resources and lastly to reduce the overall costs of care.^13^

A study by Dorr et al where 302 chest radiographs from nine different databases where randomly reported by 54 radiologists with and without the assistance of an AI algorithm for the detection of Covid-19 pneumonia. Comparison between results of radiologists with AI assistance and those without was made and was found that AI assistance increased report sensitivity by 70% from 47 to 61% but decreased specificity from 79 to 75%. The area under the curve AUROC was 0.96 for the validation set and 0.83 for the external set test, concluding that the use of AI increased physician diagnostic sensitivity for Covid-19 pneumonic changes and this may yield positive results in future allocation of resources.^16^

In a recent study by Mushtaq et al, the role of a deep-learning AI algorithm in prognostication of initial chest radiographs of Covid-19 positive patients was found to be comparable to that of a radiologists assessment score.^25^

In conjunction to a study done by Liew et al where a deep-learning model CAPE (Covid-19 AI Predictive Engine) was trained with 2337 plain chest radiographs of Covid-19 patients and was able to predict the outcome of intensive care unit patients admission and mortality with an AUC of 0.79 in comparison with clinical and laboratory data thus AI is able to predict the severity of Covid-19 disease and its outcome.^26^

A study by Bullock et al. in the year 2020 concluded that AI algorithms can be used to report Covid-19 plain chest radiographs and computed tomography images with accuracy and at a rapid speed that could save the radiologists time and use lower budgets thus saving expenses.^22^

In a study by Zhang et al, an AI algorithm CV-19 which is a net deep-learning algorithm was used to differentiate Covid-19 pneumonia in chest radiographs and compared to experienced thoracic radiologists. A sample number of 5,806 images was used to train the algorithm where 2060 images were Covid-19 pneumonia positive patients and 3148 were non-Covid-19 patients. A sample of 500 chest radiographs was then used and the AI algorithm was compared to the reports of three experienced thoracic radiologists. The overall performance sensitivity was noted to be 88% and specificity was 79% with the AUC equal to 0.92 (area under receiver operating characteristic curve). A total of randomly sampled 500 images showed the CV-19 AUC equal to 0.94 in comparison with 0.85 for experienced thoracic radiologists. Thus, CV-19 algorithm can differentiate Covid from non-Covid pneumonia better than the experienced thoracic radiologist improving the overall accuracy.^17^

Another study by Murphey et al evaluated the performance of AI in detecting Covid-19 pneumonia on chest radiography using a deep-learning algorithm CAD4COVID-XRAY, which was trained with 24,678 chest radiographs and where 454 patients were sampled of which 223 had positive PCR test and 231 were negative. The chest radiographs were evaluated for Covid-19 pneumonia by the algorithm, and the results were compared to reports of six experienced radiologists. The AI program steps include image normalization and lung segmentations by a U-net software then patch analysis by a convolutional neural network. The AI algorithm results show an 85% sensitivity and 61% specificity with an AUC of 0.81. AI has outperformed each of the radiologists at the highest possible values and at the lowest values only a single radiologist outperformed the algorithm.^19^ Nonetheless, no comparison between the radiologist reports of Covid-19 pneumonia with and without AI algorithm support was conducted.^16^

In a recent study by Zokaeinikoo et al, where a publicly available dataset of 5,801 patients was used to train an AI algorithm AIDCOV to detect abnormal chest radiography findings of Covid-19 pneumonia. There were 269 positive Covid-19 pneumonia patients and 3,949 patients with other causes of pneumonia and the rest of the 1,583 images were normal. The sample size of the study is 580. The results showed a mean cross-validation accuracy of approximately 97.8% with a sensitivity of 99.3% and specificity of 99.98% and a positive-predictive value of 99.6%. The steps used in the algorithm included image resizing, extraction of features, horizontal feature encoding and vertical feature encoding. AIDCOV can aid clinicians to the specific location of pulmonary abnormal findings rapidly and with a high sensitivity and specificity. Nevertheless, the high positive-predictive value also decreases the burden on healthcare facilities by having a lower false-positive values thus less number of patients for admission.^21^

Another study by Sukhija et al where a readily available AI algorithm NEURACOVID was used to detect Covid-19 pneumonia on 457 chest radiographs where 295 were Covid-19 positive and 162 were negative and was compared to the reporting of a single radiologist to give the report as normal, Covid-19 pneumonia or non-Covid pneumonia. The sensitivity of the radiologist reports was 44.1% and specificity was 92.6% with the AUCROC was 0.68 and positive predictive value of 91.6% while the AI program was found to have a sensitivity of 41.6% and specificity of 60% with the AUROC of 0.48 and PPV of 65%. Thus, the radiologists performance have surpassed that of the AI algorithm however the sensitivity was somewhat similar.^27^

In a recent study by wehbe et al., the deep-learning artificial algorithm DeepCOVID-XR was trained by a large data set of 5,853 patients with 300 random images used to detect Covid-19 pneumonia on plain chest radiographs in comparison with five experienced thoracic radiologists. The sensitivity was found to be 75% and specificity approximately 93% with an AUC of 0.88 which denoted similar results to that of an experienced radiologist.^28^

Machine learning is found to have a rapid and accurate application in the detection and prognostication of Covid-19 pneumonia on chest radiography and computed tomography. Extensive efforts have been forwarded globally to aid containing the Covid-19 pandemic by the use of AI and in particular machine learning.^29^

In a recent systemic review by Roberts et al where available studies and manuscripts based on the use of AI during the Covid-19 pandemic were examined noting that these studies were established on large amounts of multimodal information data from patients for the early detection and diagnosis and clinical triage according to the patients urgency. A prediction model of risk of bias assessment tool guidance (PROBAST) was used in the study and the major challenges were found to be:

- A variable international dataset.
- Considerable bias in smaller datasets.
- Difficulty in the prognostication of the disease.
- Poor integration of the imaging multistream data.
- Clinical relevance of the outcome with a highlight on the need for both clinicians and data analysts to work together.^29^

Few studies comparing the ability of AI algorithms to that of a radiologist in reporting plain chest radiographs have been implicated as being overly optimistic in performance evaluations due to the urgency of the situation of the global pandemic. Some were found to use poor quality data since the models in imaging data are limited by the quality of their specific training data, machine-learning methodology applications as well as some bias and flows in the methodology and study designs. Finally, and most importantly the low ability to reproduce the findings was found to be a major limiting factor.^29^

The use of AI has established a crucial role in healthcare applications during the Covid-19 pandemic. Nevertheless, the development of AI algorithms is complex and requires the complimentary combination of both computational and clinical expertise hand in hand with the addition of high-quality stream of healthcare data to be used for training.^13,29^
